# Exploring Host–Pathogen Interactions through Biological Control

**DOI:** 10.1371/journal.ppat.1004865

**Published:** 2015-06-25

**Authors:** Francesca Di Giallonardo, Edward C. Holmes

**Affiliations:** 1 Marie Bashir Institute for Infectious Diseases and Biosecurity, The University of Sydney, Sydney, Australia; 2 Charles Perkins Centre, The University of Sydney, Sydney, Australia; 3 School of Biological Sciences, The University of Sydney, Sydney, Australia; 4 Sydney Medical School, The University of Sydney, Sydney, Australia; University of Michigan Medical School, UNITED STATES

Invasive pests impose a major burden on the agricultural industries and the natural environment, threatening some native species with extinction. Although chemical agents are often deployed to eliminate pests, unspecific toxicity makes their use undesirable. Biological agents are of interest as an alternative method to cull specific pest species with little residual environmental harm. The deliberate release of microbial pathogens, referred to as biological control (biocontrol), has, to date, largely involved viruses. Here, we summarise the past and proposed applications of the biocontrol of vertebrate pests. Despite understandable fears, vertebrate biocontrol has yet to be associated with adverse effects, and provides large-scale natural experiments by which to understand the intimate interactions between hosts and their pathogens.

## A Short History of Biocontrol

Biocontrol, in which a biological agent is used to reduce the population size of a target pest organism, is widely used in agriculture to protect plants from arthropods and fungal infections [1]. Although biocontrol has, to date, played little role in human health, a recent and innovative application involves reducing burden of the vector-borne dengue virus (DENV) through *Aedes aegypti* mosquitoes artificially infected with *Wolbachia* bacteria [2]. While *Wolbachia* can strongly inhibit DENV in laboratory experiments, and can spread among wild mosquitoes, whether it will lead to a reduction in human DENV prevalence and incidence has yet to be determined [2,3].

**Fig 1:**
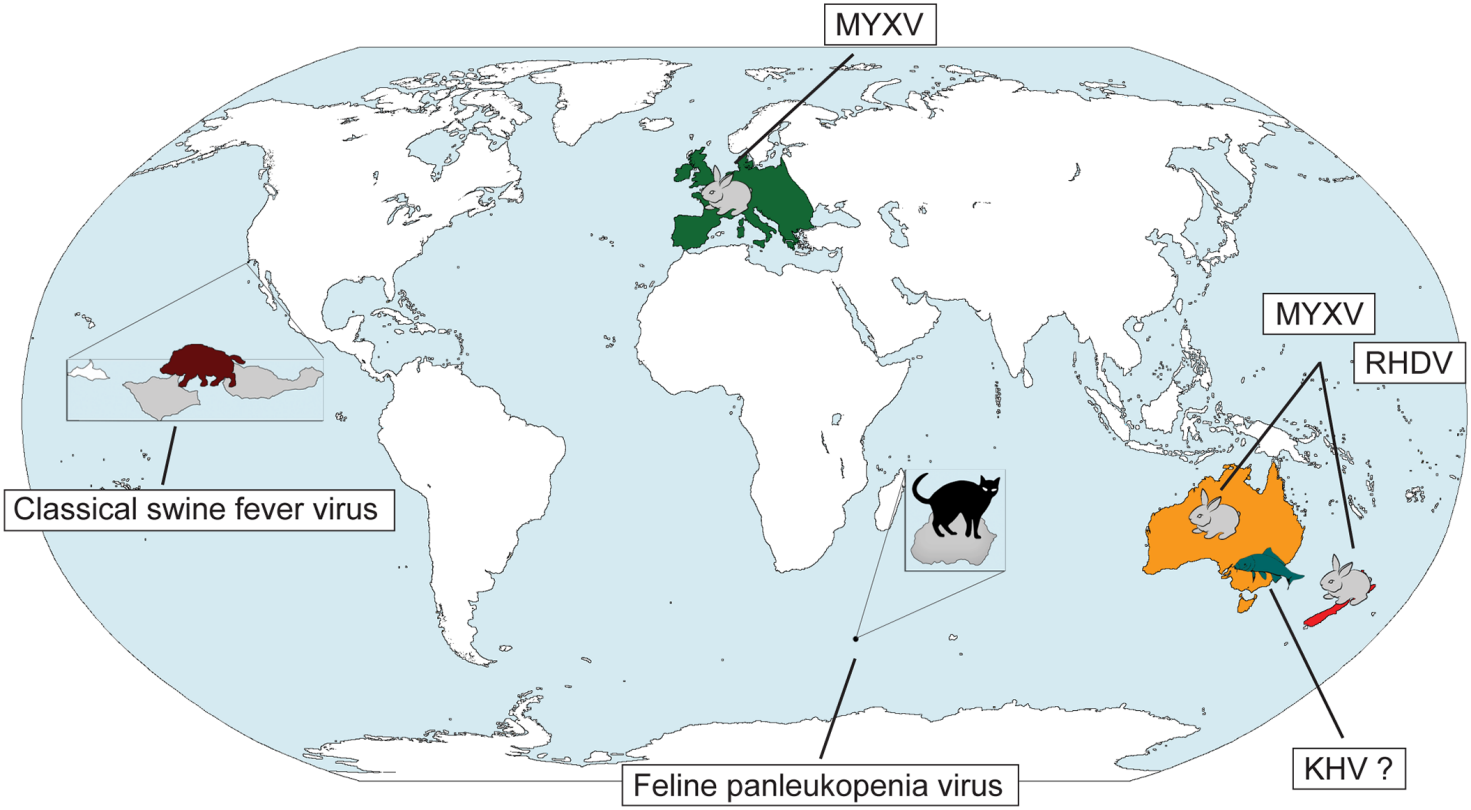
Past and future viral biocontrols of vertebrate pest species. A limited number of viruses have been used to control invasive vertebrate pests on a global scale: (i) Feline panleukopenia virus against cats on Marion Island (magnified on the map in grey), (ii) MYXV against rabbits in Europe and Australia, and (iii) RHDV in Australia and New Zealand. Despite some initial success, classical swine fever virus proved to be an unsuccessful biocontrol against wild boar on the Channel Islands in California (the islands are magnified, with the two islands affected, Santa Cruz and Santa Rosa, shown in grey). Safety testing is currently underway for the possible release of KHV against carp in Australia. Abbreviations: KHV koi herpesvirus, MYXV myxoma virus, RHDV rabbit haemorrhagic disease virus.

The use of pathogens as biocontrol agents to control vertebrate populations has caused more concern, and the fear of inadvertent infection of other species, even humans, persists. In the 1950s, classical swine fever virus was released on small islands in California in an attempt to reduce the wild swine population, but the virus did not establish persistent transmission [4]. Indeed, to date, there have only been three successful viral biocontrols of vertebrates: the release of Feline panleukopenia virus (parvovirus) to eliminate cats on Marion Island and the well-documented cases of myxoma virus (MYXV) and rabbit haemorrhagic disease virus (RHDV) in Australia and New Zealand to control the feral rabbit population (Fig 1). In all three cases an immediate reduction in the size of the target population was observed and, critically, without widespread jumping to non-target hosts. A new biocontrol intervention is planned in Australia with the release of koi herpesvirus (KHV, or cyprinid herpesvirus-3) against the common carp (*Cyprinus carpio*), an invasive fish species that comprises up to 90% of fish biomass in the major Murray-Darling river system of southeast Australia and the cause of environmental degradation and loss of native fish biodiversity [5]. If KHV is released as planned, the future study of the carp-herpesvirus system may provide insights into the nature of host—pathogen coevolution that parallel Frank Fenner’s classic work on myxomatosis in Australia [6].

## Parvovirus and the Cats of Marion Island

Marion Island is a small and remote island in the Southern Indian Ocean with a large seabird population. In 1949, cats were brought to the island to hunt mice in the island’s meteorological station. By 1975, the island had an estimated feral cat population of over 2,000, which posed a major threat to local avian species, with more than 45,000 birds predated each year. Feline parvovirus is a highly contagious (DNA) virus that is transmitted via body fluids or contaminated surfaces and resistant to temperature changes, making it ideal for a sub-Antarctic environment [7]. Following its release on Marion Island in 1977, the cat population declined to approximately 600 animals within five years, with the remaining cats successfully eradicated using traps and hunting. The island has been cat-free since 1991 [8].

## Australia: The Evolving Story of Rabbits and Viruses

Australia’s unique fauna and flora are highly sensitive to imported species, and the deliberate or accidental introduction of feral animals has posed an enormous problem to local wildlife. Historically, the most important of these has been the European rabbit (*Oryctolagus cuniculus*). Following importation for hunting purposes in the 19^th^ century, rabbits rapidly spread all across much of Australia, forming a “grey blanket” that caused major damage to vegetation [9]. Attempts to control the population, including large-scale rabbit-proof fences, failed [9], such that a new approach was clearly needed.

MYXV belongs to the family *Poxviridae* and is native to the Americas, causing only mild skin lesions in its natural lagomorph hosts (*Sylvilagus* spp.), but highly virulent in the European rabbit, resulting in myxomatosis and death within a few days [10]. In 1950, MYXV was successfully released as a biocontrol into wild rabbits in Australia, causing an immediate reduction in rabbit numbers. However, the rabbit population began to recover after a few years, reflecting a combination of evolving virus attenuation and rabbit resistance [11]. Fortuitously, a new candidate biocontrol agent—the calicivirus RHDV—was identified in a major outbreak in China in 1984, resulting in the death of 140 million rabbits in one year [12]. After its accidental spread to the Australian mainland, RHDV was officially approved as a biocontrol there in 1996, and the virus reduced the rabbit population to 5% of its former size within a few months [9]. In marked contrast to MYXV, RHDV has maintained its high virulence in rabbits, despite some resistance evolution.

Interestingly, MYXV was also released in New Zealand, but it failed to establish there, likely due to the lack of appropriate insect vectors [13]. The New Zealand government later refused the use of RHDV as a rabbit biocontrol. Nevertheless, in 1997, RHDV was illegally imported by farmers, spreading rapidly across the country and killing rabbits in a parallel manner to that observed in Australia, with a lethality rate of up to 84%. In both countries, the impact of RHDV varied among geographic regions, with lower death rates in wetter areas [9,14]. It is believed that this regional variation likely reflects cross-immunity to pre-existing rabbit caliciviruses in these localities in Australia [15].

## Rabbit Biocontrol in Europe

Although a native species, the European rabbit is regarded as a pest in some parts of Europe. Hence, two years after the successful use of MYXV in Australia, the virus was privately released as a biocontrol in France. The impact was enormous. The virus spread rapidly through France and England, reducing the size of the rabbit population by more than 90% [9]. Thirty years later, the highly virulent RHDV naturally spread through European rabbit populations. However, the overall impact of RHDV on rabbit numbers in Europe was smaller than in Australia and New Zealand, again, perhaps reflecting cross-protection from related viruses present, as well as a longer time-scale of resistance evolution [12]. Importantly, the decline in the rabbit population caused by MYXV and RHDV also had a number of negative ecological consequences. For example, in Spain, the loss of rabbits reduced food availability for their natural predators, notably the Iberian lynx [12,13].

## Insights into Virus Emergence and Evolution

The epizootics of MYXV and RHDV in Australia constitute a unique natural experiment in how viruses establish themselves in a novel and naïve host population, including the subsequent evolutionary “arms race” between hosts and pathogens and the evolution of virulence [15]. As such, they serve as a powerful analogy for natural emergence events, providing essential comparative data.

To be effective as a biocontrol, a virus needs to be species-specific, readily transmissible, and highly virulent. The breadth of host range of some viruses is so broad that they can be regarded as species “generalists,” with parvoviruses serving as an important example [16]. Indeed, the feline parvovirus used on Marion Island is a true host generalist, with a limited repertoire of mutations in the virus capsid protein enabling the infection of diverse carnivore (and perhaps other) species [17]. However, such generalists are likely rare, and most viruses have a more restricted host range, indicating that there must be important barriers to their establishment in novel species [18]. Because the host range of a pathogen is difficult to predict, and the barriers that prevent successful host jumps are largely obscure, rigorous species testing is needed before the use of any biocontrol. This is the case with both MYXV and RHDV as, to date, no inadvertent infections of species other than lagomorphs have been identified [15], although the genetic determinants of host range are unknown in both cases. Tests of species specificity are currently being performed with KHV in advance of its possible release, although Australia contains no native cyprinid fish [5].

Sustained high virulence (with high transmission intensity) is also critical to effective biocontrol. Ideally, the virus should spread quickly and kill rapidly, but retain the ability to readily transmit to new hosts. MYXV and RHDV are both highly virulent in European rabbits, killing within a few days. For reasons that are currently unclear, these viruses have experienced strikingly different trajectories of virulence evolution: the mean virulence of field strains of MYXV declined (although high virulence strains are still sampled), while there is no evidence that RHDV has attenuated [19]. Unfortunately, despite the remarkable economic and ecological benefits of both MYXV and RHDV, the rabbit population of Australia is recovering [20], which may exacerbate the decline and eventual extinction of Australia’s unique species diversity [21].

## Outlook

If performed safely, biocontrol interventions are a potentially powerful way to control invasive pest species and have provided important insights into the patterns, dynamics and processes of virus—host coevolution. We await the release of koi herpersvirus with interest.
